# Hesperidin ameliorates H_2_O_2_-induced bovine mammary epithelial cell oxidative stress via the Nrf2 signaling pathway

**DOI:** 10.1186/s40104-024-01012-9

**Published:** 2024-04-09

**Authors:** Qi Huang, Jiashuo Liu, Can Peng, Xuefeng Han, Zhiliang Tan

**Affiliations:** 1 CAS Key Laboratory for Agro-Ecological Processes in Subtropical Region, National Engineering Laboratory for Pollution Control and Waste Utilization in Livestock and Poultry Production, Hunan Provincial Key Laboratory of Animal Nutritional Physiology and Metabolic Process, Institute of Subtropical Agriculture, Chinese Academy of Sciences, Changsha, 410125 Hunan China; 2 State Key Laboratory of Animal Nutrition and Feeding, Institute of Animal Sciences, Chinese Academy of Agricultural Sciences, Beijing, 100193 China

**Keywords:** Bovine mammary epithelial cell, Hesperidin, Nrf2 signaling pathway, Oxidative stress

## Abstract

**Background:**

Hesperidin is a citrus flavonoid with anti-inflammatory and antioxidant potential. However, its protective effects on bovine mammary epithelial cells (bMECs) exposed to oxidative stress have not been elucidated.

**Results:**

In this study, we investigated the effects of hesperidin on H_2_O_2_-induced oxidative stress in bMECs and the underlying molecular mechanism. We found that hesperidin attenuated H_2_O_2_-induced cell damage by reducing reactive oxygen species (ROS) and malondialdehyde (MDA) levels, increasing catalase (CAT) activity, and improving cell proliferation and mitochondrial membrane potential. Moreover, hesperidin activated the Keap1/Nrf2/ARE signaling pathway by inducing the nuclear translocation of Nrf2 and the expression of its downstream genes *NQO1* and *HO-1*, which are antioxidant enzymes involved in ROS scavenging and cellular redox balance. The protective effects of hesperidin were blocked by the Nrf2 inhibitor ML385, indicating that they were Nrf2 dependent.

**Conclusions:**

Our results suggest that hesperidin could protect bMECs from oxidative stress injury by activating the Nrf2 signaling pathway, suggesting that hesperidin as a natural antioxidant has positive potential as a feed additive or plant drug to promote the health benefits of bovine mammary.

**Graphical Abstract:**

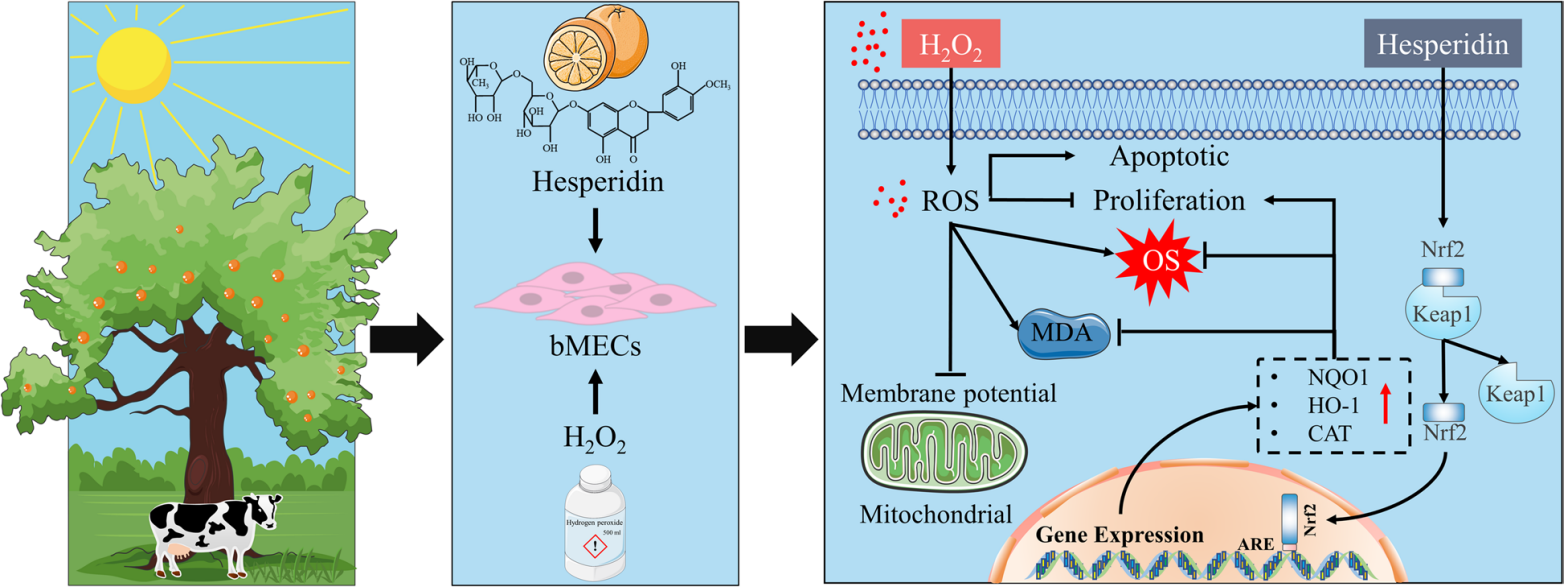

**Supplementary Information:**

The online version contains supplementary material available at 10.1186/s40104-024-01012-9.

## Introduction

Reactive oxygen species (ROS) are highly reactive and unstable molecules generated as byproducts of cellular metabolism. They can damage cellular components such as lipids, proteins, and DNA, and disrupt cellular homeostasis [1]. Bovine mammary epithelial cells (bMECs) are the main functional cells in the mammary gland that synthesize and secrete milk. ROS production is increased in bMECs during lactation due to their high metabolic rate and oxygen demand [2]. Moreover, various stress factors can also induce excessive ROS generation, such as bacterial infection, heat stress, or negative energy balance [3]. Excessive ROS can trigger cell death pathways (such as apoptosis and necrosis) and impair mammary gland function and integrity [4]. Apoptosis and oxidative stress in bMECs detrimentally affect the health and productivity of dairy cows by reducing milk yield and quality, increasing susceptibility to mastitis, and impairing reproductive performance [5].

Plant extracts, such as flavonoids, phenolic acids, terpenoids, and alkaloids, have antioxidant, anti-inflammatory, anti-apoptotic, and free radical scavenging properties [6–8]. Hesperidin is a flavonoid abundantly present in citrus fruits, such as oranges and lemons. It has been reported to have anti-inflammatory, anti-apoptotic, free radical scavenging, cholesterol-lowering, anti-allergic, anti-hypertensive, anti-carcinogenic, and anti-viral effects on various cells [9–11]. However, the protective effects of hesperidin on bMECs oxidative stress have not yet been elucidated. Previous studies have reported that hesperidin can modulate the expression of genes related to apoptosis and antioxidant response [12, 13]. For example, hesperidin alleviated oxidative stress, inflammation, and apoptosis in rat neurotoxicity or mice myocardial ischemia by regulating the B-cell lymphoma 2 (Bcl-2)/Bcl-2-associated X protein (Bax)-cysteine-aspartic acid protease 3 (caspase-3) [14] or the nuclear factor erythroid 2-related factor 2 (Nrf2) pathway [15]. We previously reported that hesperidin can inhibit hydrogen peroxide (H_2_O_2_)-induced apoptosis of bMECs by modulating the Bcl-2/Bax-caspase-3 pathway [16]. Therefore, we hypothesized that hesperidin could prevent oxidative damage in bMECs by regulating the Nrf2 pathway. The Nrf2 pathway is a major antioxidant response pathway that requires the activation of Nrf2, a transcription factor that regulates the expression of certain antioxidant enzymes, such as heme oxygenase-1 (HO-1) and NAD(P)H quinone oxidoreductase 1 (NQO1) [17].

The aim of this study was to investigate the protective effects of hesperidin on bMECs with oxidative damage caused by H_2_O_2_, a common agent for inducing oxidative stress in vitro that is widely used to establish oxidative stress models. This study could provide new insights into the pathogenesis of bovine mastitis associated with oxidative stress and the potential application of hesperidin as a natural antioxidant for bovine mammary health.

## Materials and methods

### Culture of bovine mammary epithelial cells

The bMECs were primary epithelial cells that were isolated from mammary tissue of lactating Holstein dairy cows by the Ruminant Research Group at the Institute of Subtropical Agriculture, Chinese Academy of Science (Changsha, Hunan, China) [18]. The bMECs were cultured in an incubator (8000DH, Thermo Fisher, USA) with a humidified atmosphere containing 5% CO_2_ at 37 °C in DMEM/F12 medium (Gibco, USA), supplemented with 10% (v/v) fetal bovine serum (Gibco, USA), 100 U/mL penicillin (Gibco, USA), 100 μg/mL streptomycin (Gibco, USA), 2.5 μg/mL amphotericin B (Solaibao Technology Co., Ltd., Beijing, China), 5 μg/mL hydrocortisone (Sangon Biotech Co., Ltd., Shanghai, China), 10 ng/mL epidermal growth factor (Sangon Biotech Co., Ltd., Shanghai, China), and 10 μg/mL Insulin-Transferrin-Selenium (Gibco, USA). Cells were seeded in a TC-treated multiple well plate or a culture dish (Corning, NY, USA). The medium was changed every 2 d. Cells were passaged or experiments were performed when cells reached approximately 80% confluence.

### Treatment methods for cells

Hesperidin (97% purity) was provided by Jiyuan Technology Co., Ltd. (Zhangjiajie, China). Hesperidin was dissolved in dimethyl sulfoxide (DMSO, Sigma Aldrich Corporation, St. Louis, MO, USA) at a concentration of 200 mg/mL as a stock solution for storage and diluted to specific concentrations in cell culture medium for cell treatments. Specifically, a hesperidin stock solution was freshly prepared by dissolving 200 mg of hesperidin in 1 mL of DMSO (Sigma Aldrich Corporation, St. Louis, MO, USA) and filtering it using a 0.22-μm syringe filter into a clean glass screw-top vial. The hesperidin stock solution was diluted in serum-free DMEM-F12 culture medium to concentrations of 20, 40, 60, 80, 100, 120, 140, 160, 180, and 200 μg/mL, and the same volume of DMSO was added to the control groups. The final concentration of DMSO in the treatment solutions prepared above was less than 0.1% (v/v). H_2_O_2_ (3 wt%, Sigma Aldrich Corporation, St. Louis, MO, USA) was dissolved in serum-free DMEM-F12 culture medium to a specific concentration (200, 400, 600, 800, and 1,000 μmol/L) for cell treatment. In subsequent experiments, the concentration of hesperidin was fixed at 120 μg/mL for 24 h and the concentration of H_2_O_2_ was fixed at 1,000 μmol/L to treat bMECs for 8 h.

To investigate the protective effect of hesperidin on H_2_O_2_-induced bMECs injury, the bMECs were categorized into the following groups: (1) control group (CK); (2) H_2_O_2_ model group; (3) hesperidin group; and (4) H_2_O_2_ + hesperidin group. The control and hesperidin cells were pretreated with serum-free DMEM-F12 for 8 h, and the H_2_O_2_ and H_2_O_2_ + hesperidin cells were pretreated with 1,000 μmol/L H_2_O_2_ for 8 h. Subsequently, the control and H_2_O_2_ cells were treated with serum-free DMEM-F12 for 24 h, and the hesperidin and H_2_O_2_ + hesperidin cells were treated with 120 μg/mL hesperidin for 24 h.

To investigate the role of the Nrf2 pathway in the hesperidin-mediated protective effect on H_2_O_2_-induced bMECs injury, bMECs were divided into 6 groups: (1) control group; (2) H_2_O_2_ model group; (3) H_2_O_2_ + hesperidin group; (4) ML385 (the Nrf2 inhibitor) group; (5) ML385 + H_2_O_2_ group; and (6) ML385 + H_2_O_2_ + hesperidin group: the first 3 groups were cultured with serum-free DMEM-F12 for 48 h, and the last 3 groups were treated with 5 μmol/L ML385 for 48 h. Next, the control and ML385 groups were pretreated with serum-free DMEM-F12 for 8 h, and the H_2_O_2_, H_2_O_2_ + hesperidin, ML385 + H_2_O_2_ and ML385 + H_2_O_2_ + hesperidin groups were pretreated with 1,000 μmol/L H_2_O_2_ for 8 h. Subsequently, the control, H_2_O_2_, ML385 and ML385 + H_2_O_2_ groups were treated with serum-free DMEM-F12 for 24 h, and the H_2_O_2_ + hesperidin and ML385 + H_2_O_2_ + hesperidin groups were treated with 120 μg/mL hesperidin for 24 h.

### Cell viability determination

Cell viability was determined using a Cell Counting Kit-8 (CCK-8; Beyotime Biotech. Inc., Shanghai, China). Briefly, cells were seeded into 96-well plates at a density of 4 × 10^4^ cells/well, incubated for 24 h, and then starved in serum-free DMEM-F12 for 1 h. The media were then replaced with fresh DMEM/F12 containing various concentrations of H_2_O_2_ (final concentration: 0, 200, 400, 600, 800, and 1,000 μmol/L), and the cells were exposed for various time intervals (6, 8, 12 and 24 h). Then 10 μL of CCK-8 solution was added to each well and incubated for 2 h at 37 °C in a humidified atmosphere of 5% CO_2_. The absorbance (OD) of each well was measured by using a microplate reader (Tecan, Männedorf, Switzerland) at a wavelength of 450 nm according to the manufacturer’s instructions. To explore the optimal treatment concentration of hesperidin, we assessed the cell viability of bMECs after exposure to different concentrations (0, 20, 40, 60, 80, 100, 120, 140, 160, 180, and 200 μg/mL) of hesperidin for 24 h. The duration of the hesperidin treatment was based on previous research [19–21]. The results were calculated using the percentage viability according to the following formula: Viability, % = 100 × (absorbance of treatment/absorbance of control).

### Detection of malondialdehyde (MDA) levels

The viability of bMECs treated with H_2_O_2_ at concentrations of 600, 800, and 1,000 μmol/L for 8 h, and at concentrations of 1,000 μmol/L for 12 h was approximately 60%–70%. These H_2_O_2_ treatment conditions were chosen to detect the MDA levels in bMECs. Cells were seeded into 6-well plates at a density of 1 × 10^5^ cells/well for 24 h. After recovery in serum-free DMEM-F12 for 1 h, the cells were treated with H_2_O_2_ (600, 800, and 1,000 μmol/L) for 8 h or with H_2_O_2_ at a concentration of 1,000 μmol/L for 12 h. The cells were harvested, and the cellular MDA contents were determined using a spectrophotometric diagnostic kit (Beyotime, Shanghai, China) according to the manufacturer’s instructions. The concentration of total protein in the cell sample was determined by a BCA Protein Assay kit (Beyotime, Shanghai, China). The MDA level is expressed as nmol/mg protein in relation to the cellular protein concentration.

### Flow cytometer detection of cell apoptosis

The cellular apoptosis status was detected for the bMECs treated with H_2_O_2_ at a concentration of 1,000 μmol/L for 8 h that had high MDA level. Briefly, bMECs were seeded into 6-well plates at a density of 1 × 10^5^ cells/well, incubated for 24 h, and then exposed to H_2_O_2_ to a final concentration of 1,000 μmol/L for 12 h. Subsequently, bMECs were digested with 0.25% trypsin without EDTA (Gibco, USA) and collected by centrifugation at 1,000 × *g* for 5 min at 4 °C in 1.5 mL microcentrifuge tubes, followed by washing twice with cold PBS. Then bMECs were stained with a combination of Annexin V-FITC and propidium iodide (PI) using a commercial kit (KeyGEN Biotech, Nanjing, China). After incubating them in the dark at room temperature for 10 min, the stained cells were analyzed using a flow cytometer (Beckman Coulter, USA).

### Measurement of ROS levels

The intracellular ROS content of bMECs was measured by flow cytometry using dichlorodihydrofluorescein diacetate (DCFH-DA) as a fluorescent probe. Briefly, the bMECs were divided into four groups: control (CK), H_2_O_2_, hesperidin, and H_2_O_2_ + hesperidin. Cells were plated at a density of 1 × 10^5^ cells/well for 24 h in 6-well plates. Then, the control and hesperidin cells were pretreated with serum-free DMEM-F12 for 8 h, and the H_2_O_2_ and H_2_O_2_ + hesperidin cells were pretreated with 1,000 μmol/L H_2_O_2_ for 8 h. Subsequently, the media of control and H_2_O_2_ cells were replaced with serum-free DMEM-F12 for 24 h, and the media of hesperidin and H_2_O_2_ + hesperidin cells were replaced with 120 μg/mL hesperidin for 24 h. Next, the cells were stained with 10 μmol/L DCFH-DA at 37 °C for 30 min in the dark. Cells were washed three times with serum-free media, and dihydrodichlorofluorescein (DCF) fluorescence was analyzed by an inverted fluorescence microscope (Leica DMI 3000B, Wetzlar, Germany). The percent fluorescence intensity, % = the mean fluorescence of each group/the mean fluorescence of the control group × 100.

### Measurement of MDA, GSH-Px, CAT and SOD

To measure intracellular levels of MDA, glutathione peroxidase (GSH-Px), catalase (CAT) and superoxide dismutase (SOD) in bMECs, cells were cultured in 6-well plates (1 × 10^5^ cells/well) and then treated as mentioned earlier in the Treatment methods for cells section. The activities of GSH-Px, CAT and SOD, and the content of MDA in cells were determined using spectrophotometric diagnostic kits from Beyotime Biotech. Inc., (Shanghai, China) according to the manufacturer’s protocols. The cells were harvested and suspended in PBS. The absorbance was detected at 340 nm for GSH-Px, 520 nm for CAT, 450 nm for SOD, and 532 nm for MDA using a microplate reader (Tecan, Männedorf, Swiss). The MDA level is expressed as nmol/mg protein relative to the cellular protein concentration. The levels of GSH-Px, CAT and SOD are expressed as U/g protein relative to the cellular protein concentration.

### Determination of cell proliferation

Cell proliferation of bMECs was detected using a 5-ethynyl-2'-deoxyuridine (EdU) cell proliferation assay kit (Beyotime, Shanghai, China). The bMECs were seeded in BeyoGold 35 mm confocal dishes (Beyotime, Shanghai, China) at a density of 1 × 10^5^ cells/dish, and then treated as described in the Treatment methods for cells section. After incubation, EdU solution was added to the medium at an equal volume and incubated for 2 h. The cells were washed twice with PBS, fixed in 4% paraformaldehyde for 15 min and permeabilized with 0.1% Triton X-100 for 10 min at room temperature. After washing twice with PBS, the cells were treated with 500 μL click reaction for 30 min at room temperature, incubated with 1 mL Hoechst dye for 10 min at room temperature, and then washed twice with PBS. Finally, the cells were observed using an inverted fluorescence microscope (Leica DMI 3000B, Wetzlar, Germany), and the fluorescence integrated density was analyzed with ImageJ software (1.50 version, NIH, USA).

### Measurement of mitochondrial membrane potential

The Mitochondrial membrane potential (MMP) of bMECs was assessed by JC-1 staining using a commercial kit (KeyGEN Biotech, Nanjing, China). JC-1, 5,5',6,6'-tetrachloro-1,1',3,3'-tetraethylbenzimidazolcarbocyanine iodide, is a cationic carbocyanine dye that can permeate the cell membrane and be used as a ratiometric indicator of MMP in cells. Briefly, cells were seeded in 6-well plates at a density of 1 × 10^5^ cells/well, incubated for 24 h, and then treated as described in the Treatment methods for cells section. Then, 1 mL of JC-1 working solution was added to the medium and incubated at 37 °C for 20 min. The cells were washed twice with 1 × incubation buffer and observed with an inverted fluorescence microscope (Leica DMI 3000B, Wetzlar, Germany) by detecting the fluorescence signals of JC-1 aggregates (red) and JC-1 monomers (green) at excitation/emission wavelengths of 525/590 nm for aggregates and 490/530 nm for monomers. The data are representative of at least three independent experiments.

### RNA extraction and reverse transcription quantitative real-time PCR

Total RNA was extracted from the bMECs using TRIzol reagent (Invitrogen, USA) according to the manufacturer’s instructions. The purity and concentration of the RNA were assessed using a NanoDrop 2000 spectrophotometer (Thermo Fisher Scientific, USA). In our study, the OD_260_/OD_280_ ratio of the total RNA was 1.9, which met the specified purity requirements. Then, for each sample, 1 μg of total RNA was used for reverse transcription using an Evo M-MLV RT Kit with gDNA Clean for qPCR (AGbio, Changsha, China). Specific primers for genes including *HO-1*, *Nrf2*, *NQO1*, *Keap1*, *p38 MAPK*, and *GAPDH* (glyceraldehyde 3-phosphate dehydrogenase) were designed using the primer 3.0 online tool (http://bioinfo.ut.ee/primer3) and *GAPDH* was employed as an endogenous control. The primer sequences were synthesized by Sangon Biotech. Co., Ltd. (Shanghai, China). Reverse transcription quantitative real-time PCR (RT-qPCR) was performed using the SYBR Green Premix Pro Taq HS qPCR Kit (AGbio, Changsha, China) and LightCycler 480II Real-Time PCR System (Roche Diagnostics GmbH, Mannheim, Germany). The reaction mixture contained the following components: 5.0 μL 2 × SYBR Green Pro Taq HS Premix (AGbio, Changsha, China), 1.0 μL cDNA, 0.3 μL upstream PCR primers and 0.3 μL downstream PCR primers. Nuclease-free water was added to a final volume of 10 μL. Each reaction was run in triplicate. The RT-qPCR reaction conditions consisted of an initial predegeneration step at 95 °C for 30 s, followed by 40 cycles of 95 °C for 5 s and 60 °C for 30 s. Relative quantities of expression for the genes of interest among different samples were calculated using the 2^−△△ct^ method, which compares the threshold cycle (Ct) values of the target genes and the reference gene in each sample and normalizes them to a control sample. The primers are listed in Table 1.

**Table 1:** The sequences of primers used for RT‒qPCR

Gene	Primer sequences (5'→3')	Product length, bp
*HO-1*	F: GAACGCAACAAGGAGAAC R: CTGGAGTCGCTGAACATAG	162
*Nrf2*	F: CCTCAAAGCACCGTCCTCAG R: GCTCATGCTCCTTCTGTCGT	168
*NQO1*	F: AACCAACAGACCAGCCAATC R: TCTATGGCAGCCTCCTTCAT	154
*Keap1*	F: CTGTCCTCAACCGTCTGCTC R: ATCCGCCACTCGTTTCTCTC	100
*p38 MAPK*	F: CGCCTGGCATATGTTTCTGAC R: CTCTGACACCCAAGTGGAGAC	175
*GAPDH*	F: GGCGTGAACCACGAGAAGTATAA R: CCCTCCACGATGCCAAAGT	120

*F* Forward, *R* Reverse, *HO-1* Heme oxygenase-1, *Nrf2* Nuclear factor erythroid 2-related factor 2, *NQO1* NAD(P)H quinone oxidoreductase 1, *Keap1* Kelch-like ECH-associated protein 1, *p38 MAPK* p38 mitogen-activated protein kinase, *GAPDH* Glyceraldehyde-3-phosphate dehydrogenase

### Protein extraction and western blotting

The total proteins of bMECs were extracted with cell lysis buffer (Beyotime, Shanghai, China). The concentration of the extracted protein was determined by the bicinchoninic acid (BCA) method (Beyotime, Shanghai, China). The protein sample was mixed with the loading buffer and then heated in a water bath at 95 °C for 10 min. Then the protein was subjected to 10% sodium dodecyl sulfate-polyacrylamide gel electrophoresis (SDS-PAGE). The separated proteins were transferred to a polyvinylidene difluoride (PVDF) membrane using the Transfer Cell (Liuyi Biotechnology, Beijing, China). The membranes were washed three times with Tris-buffered saline with 0.1% Tween 20 (TBST) buffer for 10 min each and then blocked with Quick Block Blocking Buffer (Beyotime, Shanghai, China) for 1 h. After washing three times with TBST for 15 min each, the membranes were incubated with primary antibodies at 4 °C overnight. Anti-Nrf2 (1:4,000, Cat. No. 16396-1-AP), anti-NQO1 (1:2,000, Cat. No. 11451-1-AP), anti-HO-1 (1:1,000, Cat. No. 10701-1-AP), anti-Keap1 (1:4,000, Cat. No. 10503-2-AP), anti-p38-MAPK (1:1,000, Cat. No. 14064-1-AP), and anti-β-actin (1:5,000, Cat. No. 66009-1-Ig) antibodies were purchased from Proteintech (Wuhan, China). The following day, the membrane was incubated for an additional 2 h with horseradish peroxidase (HRP)-conjugated secondary antibody (1:5,000 dilution) at room temperature after being thoroughly washed three times with TBST for 10 min each. HRP-conjugated Affinipure Goat Anti-Rabbit IgG (H + L) (SA00001-2) and HRP-conjugated Affinipure Goat Anti-Mouse IgG (H + L) (SA00001-1) were purchased from Proteintech (Wuhan, China). Finally, immunoreactive bands were visualized using an electrochemiluminescence (ECL) kit (Beyotime, Shanghai, China) and the intensity of the protein bands was assayed by Quantity One software (Quantity One 1-D Analysis Software).

### Immunofluorescence assay

The aim of the immunofluorescence assay was to detect Nrf2 protein localization in bMECs. Briefly, cells were seeded at a 1 × 10^5^ cell density per well for 24 h in 6-well plates and then treated as mentioned earlier in the Treatment methods for cells section. After washing three times with PBS, the cells were fixed with 4% paraformaldehyde for 10 min and then permeabilized with 0.1% Triton X-100 for 10 min at room temperature. After washing three times with PBS for 10 min each, the cells were incubated with the primary antibody (anti-Nrf2; Proteintech, China) at 4 °C overnight. Then the cells were washed three times with PBS and were incubated in the secondary antibody (goat anti-rabbit IgG [H + L] Alexa Fluor-488, 33106ES60, Yeasen, Shanghai, China) for 2 h at room temperature protected from light. After washing three times with PBS, the cells were incubated with DAPI solution for nuclear staining at room temperature for 15 min in the dark. Finally, the fluorescence of the cells was observed under an inverted fluorescence microscope (Leica DMI 3000B; Germany).

### Statistical analysis

Each experiment was repeated three times. The analysis of experimental results was performed using either GraphPad Prism 8.0 (GraphPad Software; San Diego, CA, USA) or SPSS 26.0 software (IBM; USA). Unless stated otherwise, all data was presented as the means ± standard error of the mean (SEM). Comparisons among groups were analyzed using one-way ANOVA followed by Tukey’s tests. Comparisons between two groups were performed using Student’s *t*-test. *P* < 0.05 was considered statistically significant. The symbol “*” was used to indicate *P* < 0.05; “**” was used to indicate *P* < 0.01, and “***” was used to indicate *P* < 0.001.

## Results

### Construction of an H_2_O_2_-induced oxidative stress model in bMECs

**Fig. 1:**
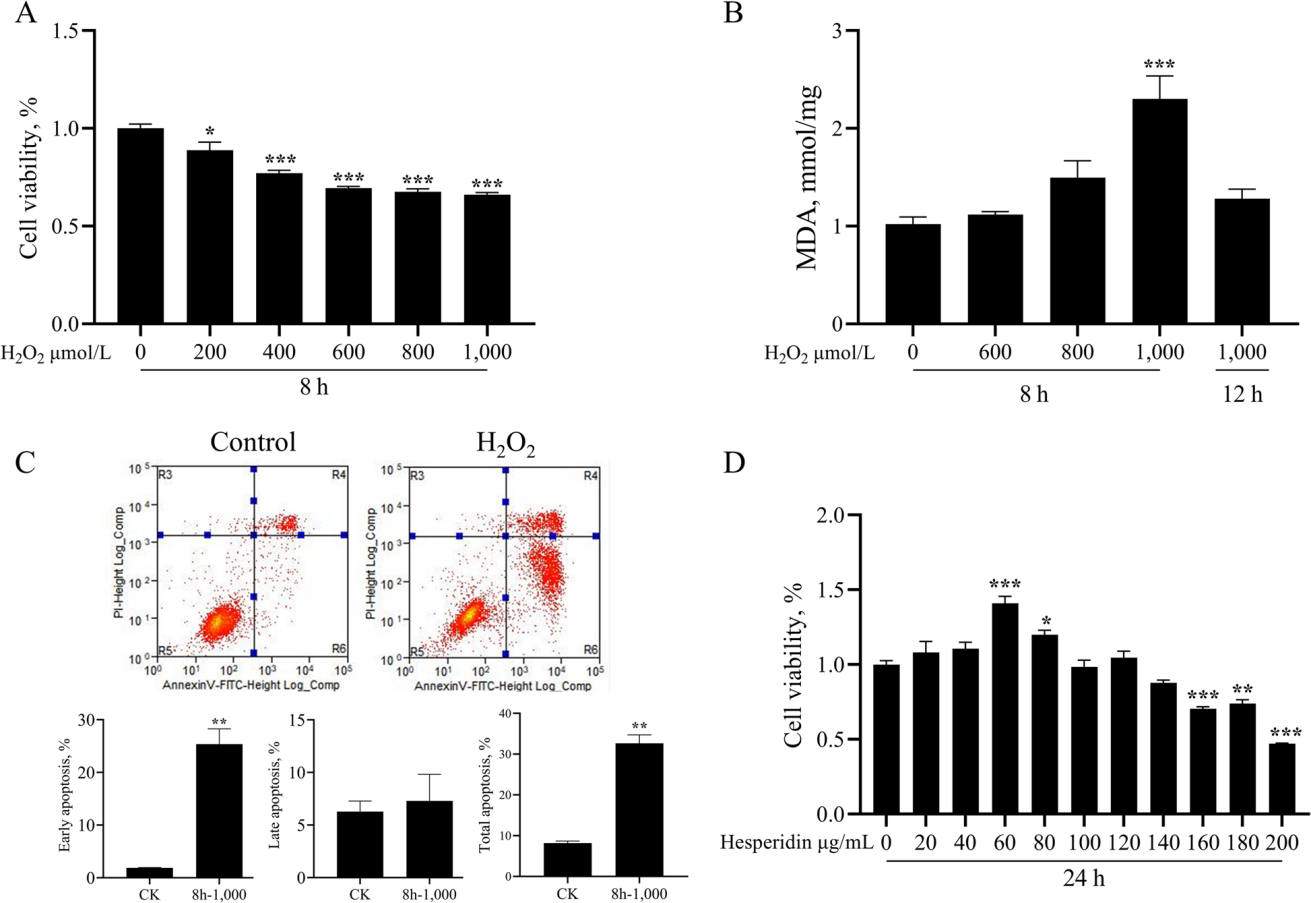
Effects of hesperidin and H_2_O_2_ on the cell viability, MDA levels and apoptosis of bovine mammary epithelial cells (bMECs). **A** Cell viability of bMECs. bMECs were treated with H_2_O_2_ (0, 200, 400, 600, 800, 1,000 μmol/L) for 8 h. **B** Malondialdehyde (MDA) content in bMECs. bMECs were treated with H_2_O_2_ (0, 600, 800 μmol/L) for 8 h and 1,000 μmol/L for 12 h. **C** The presence of apoptotic cells was detected by flow cytometry. Representative images and quantification of the early apoptosis, late apoptosis and total apoptotic rate of bMECs treated with H_2_O_2_ (1,000 μmol/L) for 8 h. **D** Cell viability of bMECs. bMECs were treated with hesperidin (0, 20, 40, 60, 80, 100, 120, 140, 160, 180, and 200 μg/mL) for 24 h. All data are presented as the mean ± SEM from three independent experiments. ^*^P < 0.05, ^**^P < 0.01, and ^***^P < 0.001

To determine the appropriate H_2_O_2_ concentration and conditions for constructing an oxidative stress model, bMECs were treated with graded concentrations of H_2_O_2_ (0, 200, 400, 600, 800, and 1,000 μmol/L) for 4 different time points (6, 8, 12, and 24 h). The CCK-8 results showed that cell proliferation decreased in a dose-dependent manner (Fig. 1A and Fig. S1). The results showed that the viability of bMECs exposed to 600, 800 and 1,000 μmol/L H_2_O_2_ for 8 h or 1,000 mol/L H_2_O_2_ for 12 h were about 60%–70%. Therefore, these treatment conditions were selected to determine the MDA content in the cells after H_2_O_2_ exposure. Treatment with 1,000 μmol/L H_2_O_2_ for 8 h significantly increased the MDA content in bMECs (*P* < 0.001, Fig. 1B). Meanwhile, this treatment also significantly increased the proportion of early apoptotic cells and the total proportion of apoptotic cells (*P* < 0.01, Fig. 1C). Hence, we selected 1,000 μmol/L H_2_O_2_ to stimulate bMECs for 8 h to establish an oxidative stress model.

### The cytotoxicity of hesperidin against bMECs and selection of optimal hesperidin concentration for protecting bMECs from oxidative stress

To demonstrate the toxic effect of hesperidin on bMECs, we first examined the dose effects of hesperidin (0, 20, 40, 80, 100, 120, 140, 160, 180, and 200 μg/mL) on bMECs viability via a CCK-8 assay. Hesperidin did not display cytotoxicity against the bMECs at increasing concentrations from 0 to 140 μg/mL with 20 μg/mL increments. The viability of cells treated with 120 μg/mL hesperidin was higher than that of cells treated with 140 μg/mL hesperidin. Compared with the control cells (0 μg/mL hesperidin), hesperidin at 60 and 80 μg/mL significantly increased the viability of bMECs (*P* < 0.05, Fig. 1D). However, hesperidin at 160 μg/mL or higher showed a toxic effect on the viability of bMECs (*P* < 0.05, Fig. 1D). Therefore, in this study, after taking into account the impact of hesperidin on bMECs viability and the potential concentration dependence of bioactive components of hesperidin, we finally chose a concentration of 120 μg/mL hesperidin for all subsequent experiments to investigate its protective effect against oxidative stress induced by H_2_O_2_.

### Hesperidin reduces H_2_O_2_-induced oxidative stress in bMECs

**Fig. 2:**
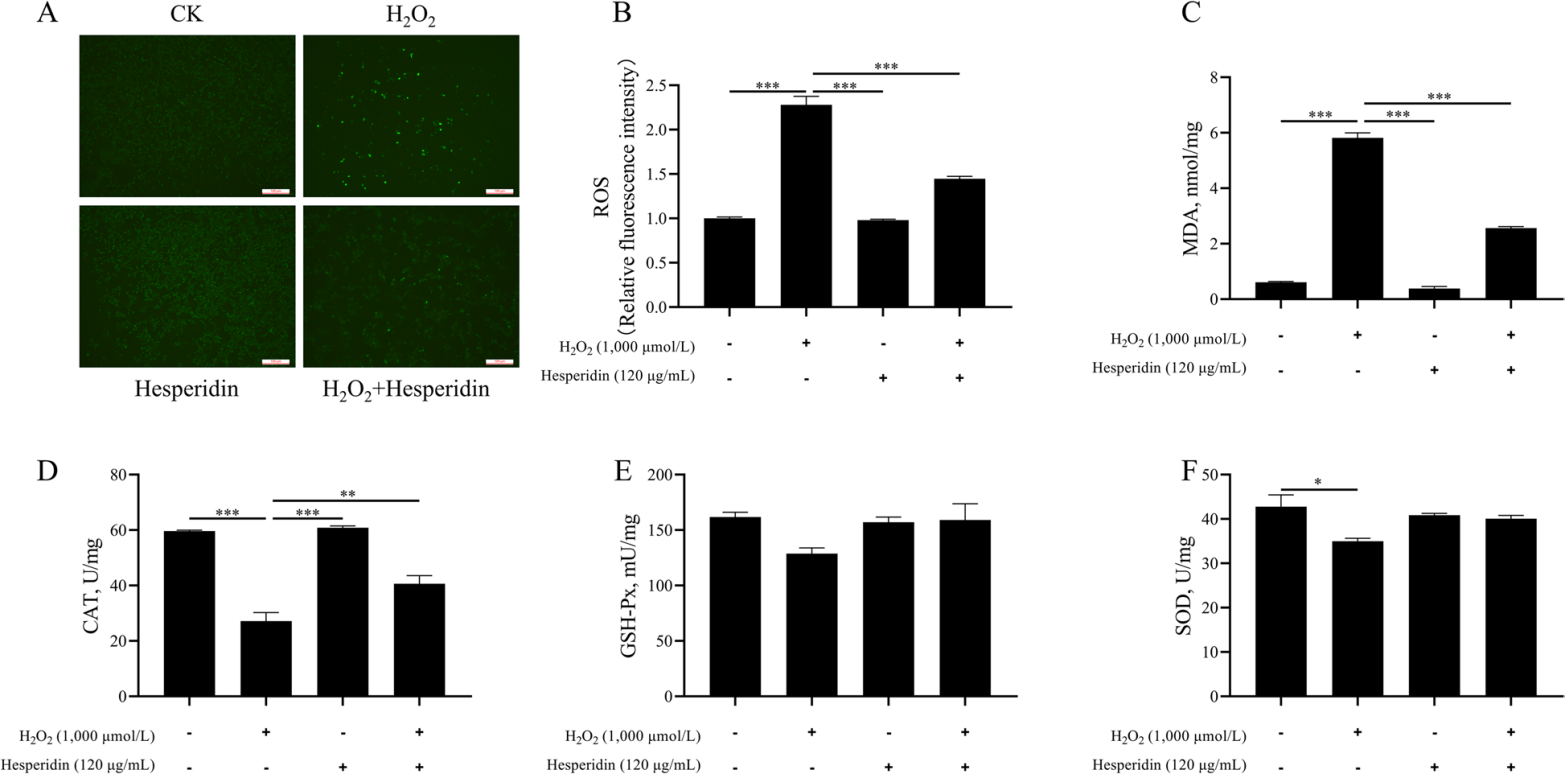
Hesperidin ameliorates H_2_O_2_-induced oxidative stress in bMECs. bMECs were treated with H_2_O_2_ (1,000 μmol/L) for 8 h and/or hesperidin (120 μg/mL) for 24 h. **A** Representative images of ROS content, the scale bar represents 100 μm. **B** ROS content. **C** Malondialdehyde (MDA) content in bMECs. **D** Catalase (CAT) content in bMECs. **E** Glutathione peroxidase (GSH-Px) content in bMECs. **F** Superoxide dismutase (SOD) content in bMECs. All data are presented as the mean ± SEM from three independent experiments. ^*^P < 0.05, ^**^P < 0.01, and ^***^P < 0.001

The purpose of this experiment was to investigate whether hesperidin can effectively alleviate the oxidative stress caused by H_2_O_2_. The results indicated that compared with the CK group, H_2_O_2_ increased the levels of ROS and MDA, and induced oxidative stress (*P* < 0.001, Fig. 2A–C). Meanwhile, compared with the CK group, H_2_O_2_ decreased the levels of CAT (*P* < 0.001) and SOD (*P* < 0.05) in bMECs and impaired their antioxidant capacity (Fig. 2D and F). However, compared with the H_2_O_2_-treated group, hesperidin significantly reduced the levels of ROS and MDA induced by H_2_O_2_ (*P* < 0.001, Fig. 2A–C) and effectively blocked the reductions in CAT (*P* < 0.01, Fig. 2D). In a similar fashion, SOD and GSH-Px activities were induced by hesperidin compared to H_2_O_2_ treatment alone although the difference in SOD and GSH-Px between H_2_O_2_ group and H_2_O_2_ + hesperidin group did not reach statistical significance (*P* > 0.05, Fig. 2E and F).

### Hesperidin improves cell proliferation and mitochondrial function in H_2_O_2_-treated bMECs

**Fig. 3:**
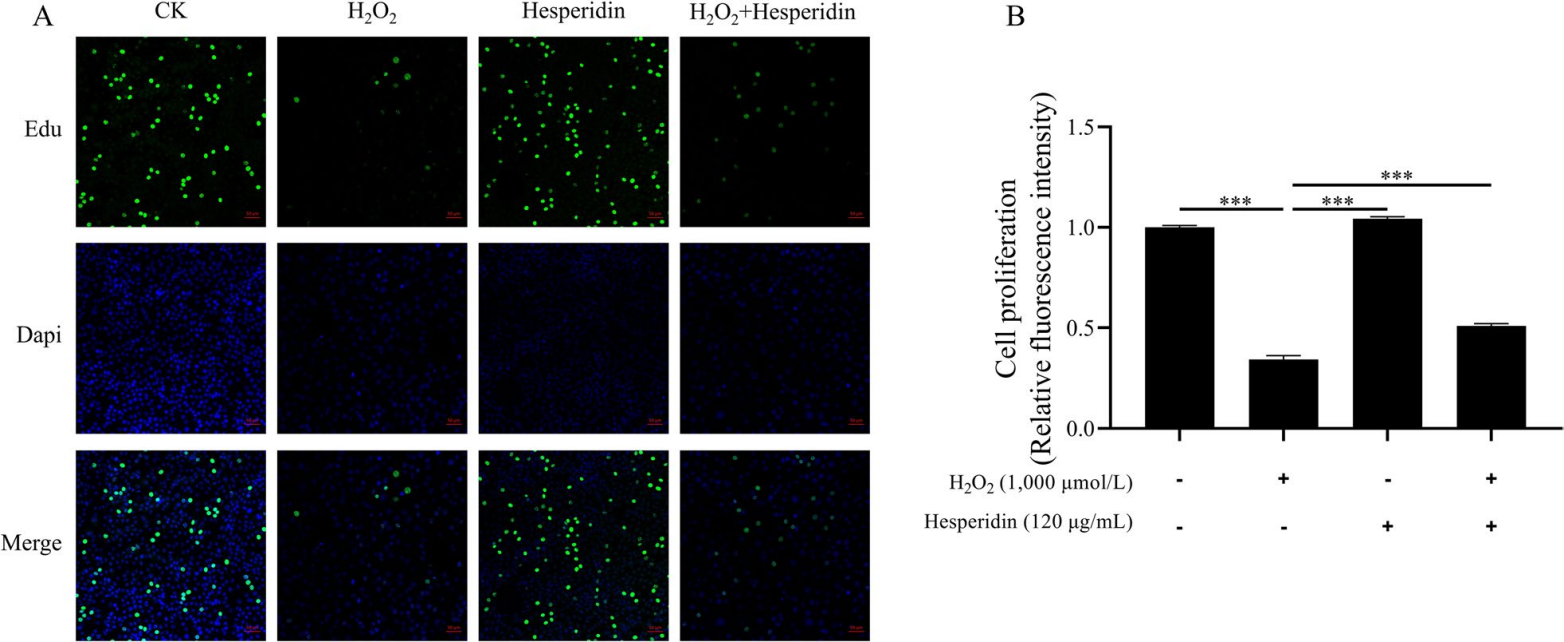
Hesperidin increases cell proliferation in H_2_O_2_-treated bMECs. **A** Representative images presenting a cell proliferation by EdU assay. The scale bar represents 50 μm. **B** Quantitative analysis of an EdU assay for cell proliferation. bMECs were treated with H_2_O_2_ (1,000 μmol/L) for 8 h and/or hesperidin (120 μg/mL) for 24 h. All data are presented as the mean ± SEM from three independent experiments. ^*^P < 0.05, ^**^P < 0.01, and ^***^P < 0.001

**Fig. 4:**
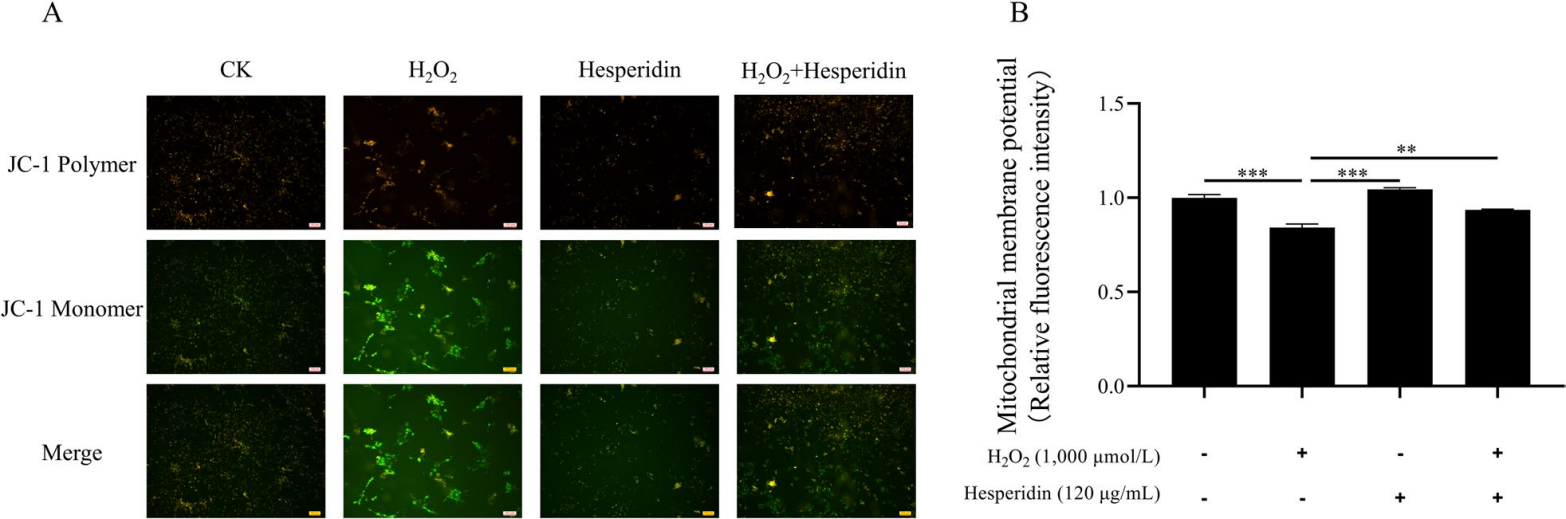
Hesperidin increases mitochondrial membrane potential in H_2_O_2_-treated bMECs. **A** Representative images presenting the mitochondrial membrane potential. The scale bar represents 100 μm. **B** Quantitative analysis of mitochondrial membrane potential. bMECs were treated with H_2_O_2_ (1,000 μmol/L) for 8 h and/or hesperidin (120 μg/mL) for 24 h. All data are presented as the mean ± SEM from three independent experiments. ^*^P < 0.05, ^**^P < 0.01, and ^***^P < 0.001

This experiment was designed to examine the role of hesperidin in protecting the proliferation of bMECs that were damaged by H_2_O_2_. We observed that H_2_O_2_ reduced the proliferation of bMECs (*P* < 0.001, Fig. 3). However, hesperidin significantly rescued the cell proliferation of bMECs that were impaired by H_2_O_2_ (*P* < 0.001, Fig. 3). Next, we used the JC-1 fluorescent probe to measure the mitochondrial membrane potential changes of bMECs under different treatments. Figure 4A and B shows that the MMP value of the H_2_O_2_ + hesperidin group was significantly higher than that of the H_2_O_2_ group. This indicates that hesperidin can reverse the damage of cell mitochondria caused by H_2_O_2_.

### Hesperidin activated the Keap1/Nrf2/ARE signaling pathway in H_2_O_2_-treated bMECs

**Fig. 5:**
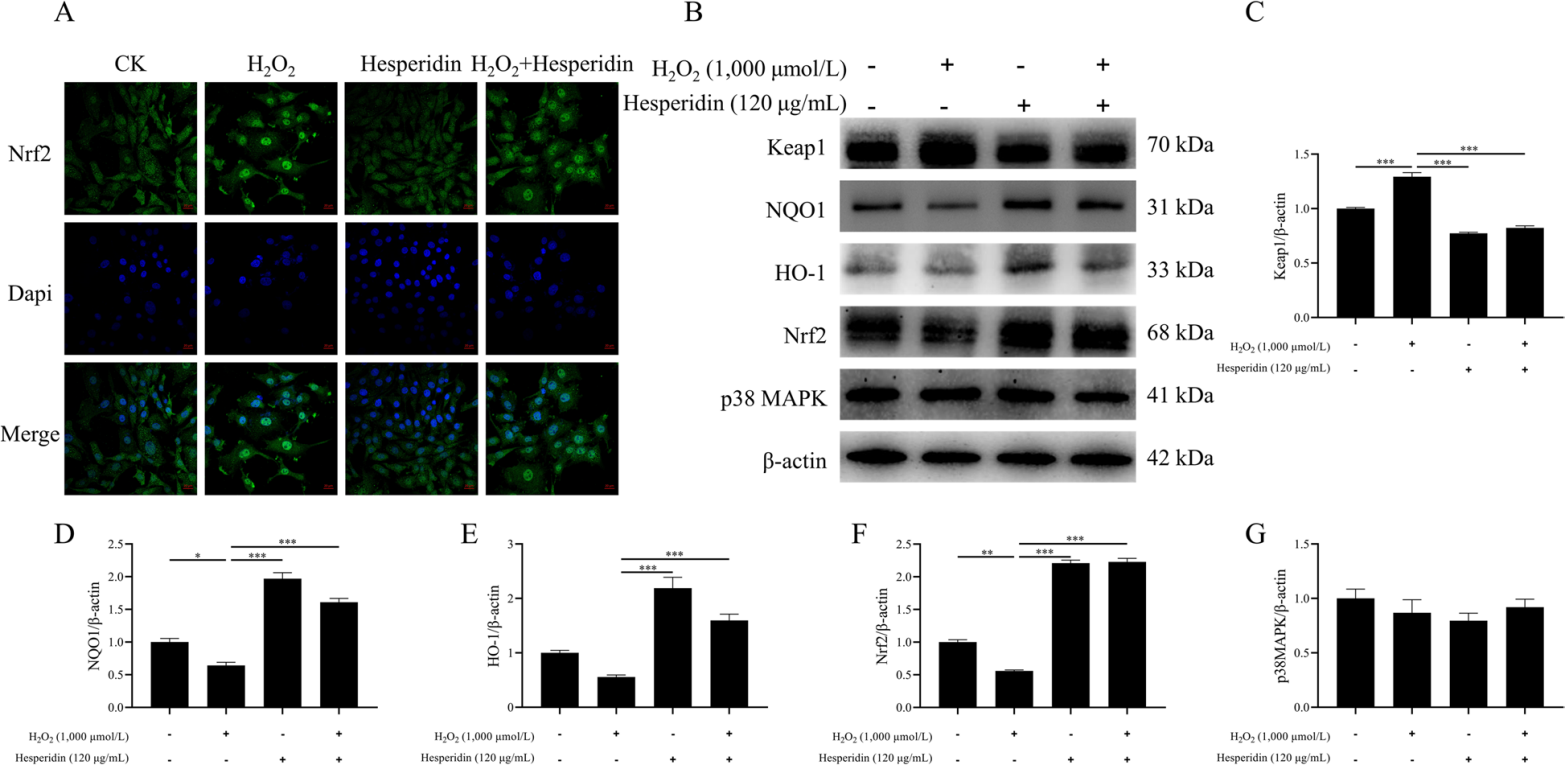
Hesperidin induced the nuclear translocation of Nrf2 and activated the expression of antioxidant response genes in H_2_O_2_-treated bMECs. **A** Representative images of the cellular distribution of Nrf2, the scale bar represents 20 μm. **B** Representative western blots of Keap1, NQO1, HO-1, Nrf2, p38 MAPK, and β-actin was used as a loading control. **C**–**G** Quantitation of Keap1, NQO1, HO-1, Nrf2, and p38 MAPK protein abundance. bMECs were treated with H_2_O_2_ (1,000 μmol/L) for 8 h and/or hesperidin (120 μg/mL) for 24 h. All data are presented as the mean ± SEM from three independent experiments. ^*^P < 0.05, ^**^P < 0.01, and ^***^P < 0.001

The purpose of this experiment was to explore whether hesperidin protects bMECs from oxidative stress by activating the Keap1/Nrf2/ARE signaling pathway and promoting the mRNA and protein expression of Nrf2 downstream genes. First, we investigated whether H_2_O_2_ and hesperidin affect the cellular distribution of Nrf2. The results showed that hesperidin can induce the nuclear translocation of Nrf2, which may activate the expression of antioxidant response genes (Fig. 5A). Then, we detected the mRNA and protein expression levels of genes associated with the antioxidation ability of bMECs. We found that H_2_O_2_ inhibited the activation of the Nrf2 signaling pathway, decreased the mRNA and protein expression of NQO1 (*P* < 0.05, Fig. 5D and Fig. S2B), HO-1 (*P* < 0.05, Fig. 5E and Fig. S2C) and Nrf2 (*P* < 0.05, Fig. 5F and Fig. S2A), and increased the expression of Keap1 (*P* < 0.001, Fig. 5C and Fig. S2D), leading to a decrease in cellular antioxidant capacity. However, hesperidin counteracted the inhibitory effect of H_2_O_2_ on Nrf2 signaling, increased the mRNA and protein expression of NQO1, HO-1 and Nrf2, decreased Keap1 expression, and restored cellular antioxidant capacity (*P* < 0.05, Fig. 5B–F and Fig. S2A–D). Interestingly, there was no significant difference in p38 MAPK mRNA and protein expression between all the groups (Fig. 5G and Fig. S2E). This suggests that the activation of the Keap1/Nrf2/ARE signaling pathway by hesperidin is independent of the p38 MAPK pathway in bMECs under oxidative stress.

### Hesperidin rescued H_2_O_2_-elicited Keap1/Nrf2/ARE signaling pathway activity through Nrf2

**Fig. 6:**
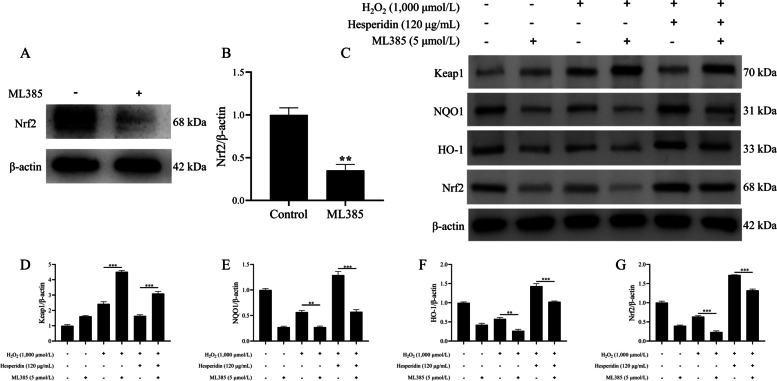
Nrf2 inhibitor blocked the activation effect of hesperidin on the Keap1/Nrf2/ARE signaling pathway. **A** Protein levels of Nrf2 and β-actin by western blot analysis. **B** Quantitation of Nrf2 protein abundance. **C** Protein levels of Keap1, NQO1, HO-1 and Nrf2 by western blot analysis. **D**–**G** Quantitation of Keap1, NQO1, HO-1, and Nrf2 protein abundance. β-actin was used as an internal control. bMECs were treated with ML385 (5 μmol/L) for 48 h and/or H_2_O_2_ (1,000 μmol/L) for 8 h and/or hesperidin (120 μg/mL) for 24 h. All data are presented as the mean ± SEM from three independent experiments. ^*^P < 0.05, ^**^P < 0.01, and ^***^P < 0.001

To verify whether the activation effect of hesperidin on the Keap1/Nrf2/ARE signaling pathway is Nrf2 dependent, ML385, a specific inhibitor of Nrf2, was used to inhibit Nrf2 expression. As shown in Fig. 6A and B, the protein level of Nrf2 was markedly decreased by treatment with ML385 compared with the control group (*P* < 0.01). Importantly, inhibition of Nrf2 significantly blocked the hesperidin-induced upregulation of Nrf2, HO-1 and NQO1 in H_2_O_2_-treated bMECs (*P* < 0.01, Fig. 6C and E–G). Meanwhile, ML385 significantly increased Keap1 expression in H_2_O_2_-treated bMECs (*P* < 0.001, Fig. 6D).

## Discussion

The mammary gland is an exocrine gland that produces milk and bMECs are the main cell type in the mammary gland of the dairy cow. In addition to their role in milk production, bMECs are also effector cells in mammary immunity [22, 23]. However, bMECs in high-yielding dairy cows are prone to oxidative stress due to their high metabolic rate [24]. Moreover, the bovine mammary gland experiences oxidative stress during the transition period. Oxidative stress can increase the level of ROS in cells, disrupt the cellular redox balance and lead to endoplasmic reticulum stress, mitochondrial dysfunction, lipid peroxidation and cell apoptosis [25, 26]. Therefore, it is important to explore effective antioxidant strategies to protect bMECs from oxidative damage and maintain bovine mammary health and lactation performance. Hesperidin is a bioflavonoid found in citrus fruits with antioxidant and anti-inflammatory properties [27]. The aim of this study was to investigate the protective effect of hesperidin on H_2_O_2_-induced oxidative stress injury in bMECs and the possible molecular mechanism. This study confirmed that hesperidin exerted antioxidant effects on bMECs.

ROS and MDA are important markers of oxidative stress, which reflect the production of free radicals and lipid peroxidation in cells [28]. Oxidative stress can cause cell dysfunction and death [29]. Therefore, reducing ROS and MDA levels is an effective way to prevent oxidative damage. Hesperidin has been shown to scavenge free radicals and inhibit lipid peroxidation [30]. In this study, we found that hesperidin could reduce the ROS and MDA levels induced by H_2_O_2_ in bMECs compared with the control group. Moreover, hesperidin increased the CAT level but had no effect on the SOD and GSH-Px levels in bMECs exposed to H_2_O_2_. This is different from previous research results. Ansar et al. found that hesperidin could reduce the damage of nano-zinc oxide to liver cells by lowering MDA levels and increasing GSH levels and antioxidant enzyme (SOD, CAT, and GSH-Px) activity [31]. This may be due to the different scavenging efficiencies of hesperidin on different types of free radicals, or the differences in the regulation of antioxidant pathways by hesperidin in various cell types. Studies have shown that hesperidin has a higher scavenging efficiency for DPPH and ABTS free radicals but a lower scavenging efficiency for superoxide anion and hydroxyl free radicals [32]. As a common reagent for inducing oxidative stress in vitro, H_2_O_2_ mainly produces hydroxyl free radicals. Hesperidin may selectively regulate different signaling pathways, such as Nrf2/ARE, NF-κB and PI3K/Akt/FOXO3, to protect cells from oxidative stress caused by different free radicals [33–35].

Cell proliferation and mitochondrial membrane potential are important indicators of cell viability and function [36, 37]. H_2_O_2_ is a common source of oxidative stress that can cause cell damage and death by inducing DNA damage, lipid peroxidation, protein oxidation and mitochondrial dysfunction [38, 39]. Our study demonstrated that hesperidin could improve the cell proliferation and mitochondrial membrane potential of bMECs exposed to H_2_O_2_. This is similar to the results of previous studies, which showed that hesperidin can prevent oxidative damage to cellular components and restore mitochondrial function [40]. However, several studies have found that hesperidin seems to affect the expression of cell cycle- and apoptosis-related genes by inducing the endoplasmic reticulum stress pathway, regulating the immune response-related or PI3K/Akt/mTOR signaling pathway, and inducing apoptosis and autophagy in various cancer cells, thereby inhibiting the proliferation of cancer cells [41–43]. This suggests that hesperidin may have different mechanisms of action on normal cells and cancer cells, and can affect different cell fates and signaling pathways, and there may be a dose–effect relationship. The specific mechanisms of action and dose–effect relationship need to be further explored.

In addition, our results indicated that hesperidin may play a protective role in bMECs through the Keap1/Nrf2/ARE signaling pathway. The Keap1/Nrf2/ARE signaling pathway is a key regulator of cellular antioxidant defense [44, 45]. Keap1 is a negative regulator of Nrf2 that binds to Nrf2 and retains it in the cytoplasm [46]. Nrf2 is a transcription factor that binds to the antioxidant response element (ARE) and activates the expression of various antioxidant genes, such as HO-1 and NQO1 [47–49]. HO-1 is an enzyme that catalyzes the degradation of heme, a pro-oxidant molecule known as biliverdin, carbon monoxide and iron, which have anti-inflammatory, anti-apoptotic and cytoprotective effects [50]. NQO1 can reduce quinones to hydroquinone, which can be further conjugated with glutathione and excreted from the cells [51, 52]. In our study, hesperidin induced the nuclear translocation of Nrf2 by disrupting the interaction between Nrf2 and Keap1 and increased the expression of HO-1 and NQO1 in H_2_O_2_-treated bMECs. According to previous studies, plant-derived compounds, such as sulforaphane, 6-methylsulfinylhexyl isothiocyanate and curcumin, could induce the activation of Nrf2 nuclear translocation [53–55]. These results are consistent with previous studies that have shown that hesperidin can protect against oxidative stress-induced cell damage by upregulating HO-1 expression via ERK/Nrf2 signaling and/or by increasing CAT and SOD activities [21, 56, 57]. However, some studies have also reported that hesperidin can modulate other signaling pathways in different cell types or under different stress conditions, including the transcription factors Foxo1 and Foxo33 or p38 MAPK, which may also be involved in the antioxidant response [58–60]. It has been reported that the MAPK pathways participate in Nrf2-dependent nuclear translocation through kinases such as ERK, JNK and p38 MAPK in response to various stimuli. Chen et al. found that hesperidin dose-dependently facilitated the phosphorylation of ERK1/2, but not the p38 and JNK pathways [56]. In our present study, we also found that this effect was Nrf2-dependent, but independent of the p38MAPK pathway. Therefore, further studies are needed to elucidate the precise mechanisms by which hesperidin regulates oxidative stress and antioxidant defense in bMECs.

## Conclusion

In conclusion, based on our results, this study found that hesperidin effectively protected against H_2_O_2_-induced oxidative damage in bMECs by inhibiting cell apoptosis, ROS overproduction and MDA formation, as well as enhancing the levels of CAT. The mechanism of action includes activating the Keap1/Nrf2/ARE pathway. Hesperidin induced the nuclear translocation of Nrf2 and the dissociation of Nrf2 from Keap1 and upregulated the expression of its downstream genes NQO1 and HO-1. Interestingly, we also found that the antioxidant effect of hesperidin was Nrf2-dependent and independent of the p38 MAPK pathway. This study provides an in vitro test basis for the application of hesperidin in the prevention and treatment of bovine oxidative stress injury in animal husbandry.

### Supplementary Information

**Additional file 1:** **Fig. S1. **Effects of H_2_O_2_ on the viability of bMECs. **A** bMECs were treated with H_2_O_2_ (0, 200, 400, 600, 800, 1,000 μmol/L) for 6 h. **B** bMECs were treated with H_2_O_2_ (0, 200, 400, 600, 800, 1,000 μmol/L) for 12 h. **C** bMECs were treated with H_2_O_2_ (0, 200, 400, 600, 800, 1,000 μmol/L) for 24 h. All data are presented as the mean ± SEM from three independent experiments. ^*^P< 0.05, ^**^P< 0.01, and ^***^P< 0.001.

**Additional file 2:** **Fig. S2.** Hesperidin induces the nuclear translocation of Nrf2 and activates the mRNA expression of antioxidant response genes in H_2_O_2_-treated bMECs. **A** *Nrf2* mRNA expression. **B** *NQO1* mRNA expression. **C** *HO-1* mRNA expression. **D** *Keap1* mRNA expression. **E** *p38 MAPK* mRNA expression. bMECs were treated with H_2_O_2_ (1,000 μmol/L) for 8 h and/or hesperidin (120 μg/mL) for 24 h. All data are presented as the mean ± SEM from three independent experiments. ^*^P< 0.05, ^**^P< 0.01, and ^***^P< 0.001.

## Data Availability

The datasets used during the current study are available from the corresponding author on reasonable request.

## Abbreviations

ARE: Antioxidant response element
Bax: Bcl-2-associated X protein
Bcl-2: B-cell lymphoma 2
bMECs: Bovine mammary epithelial cells
caspase-3: Cysteine-aspartic acid protease 3
CAT: Catalase
DCFH-DA: 2,7-Dichlorodihydrofluorescein diacetate
GAPDH: Glyceraldehyde 3-phosphate dehydrogenase
GSH-Px: Glutathione peroxidase
H_2_O_2_: Hydrogen peroxide
HO-1: Heme oxygenase-1
Keap1: Kelch-like ECH-associated protein 1
MDA: Malondialdehyde
NQO1: NAD(P)H quinone oxidoreductase 1
Nrf2: Nuclear factor erythroid 2-related factor 2
p38MAPK: P38 Mitogen-activated protein kinase
ROS: Reactive oxidative stress
SOD: Superoxide dismutase
