# Supplementary figures and images for: Hesperidin ameliorates H2O2-induced bovine mammary epithelial cell oxidative stress via the Nrf2 signaling pathway
Source: J Anim Sci Biotechnol. 2024 Apr 9;15:57. doi: 10.1186/s40104-024-01012-9 (PMC11003082; doi:10.1186/s40104-024-01012-9)

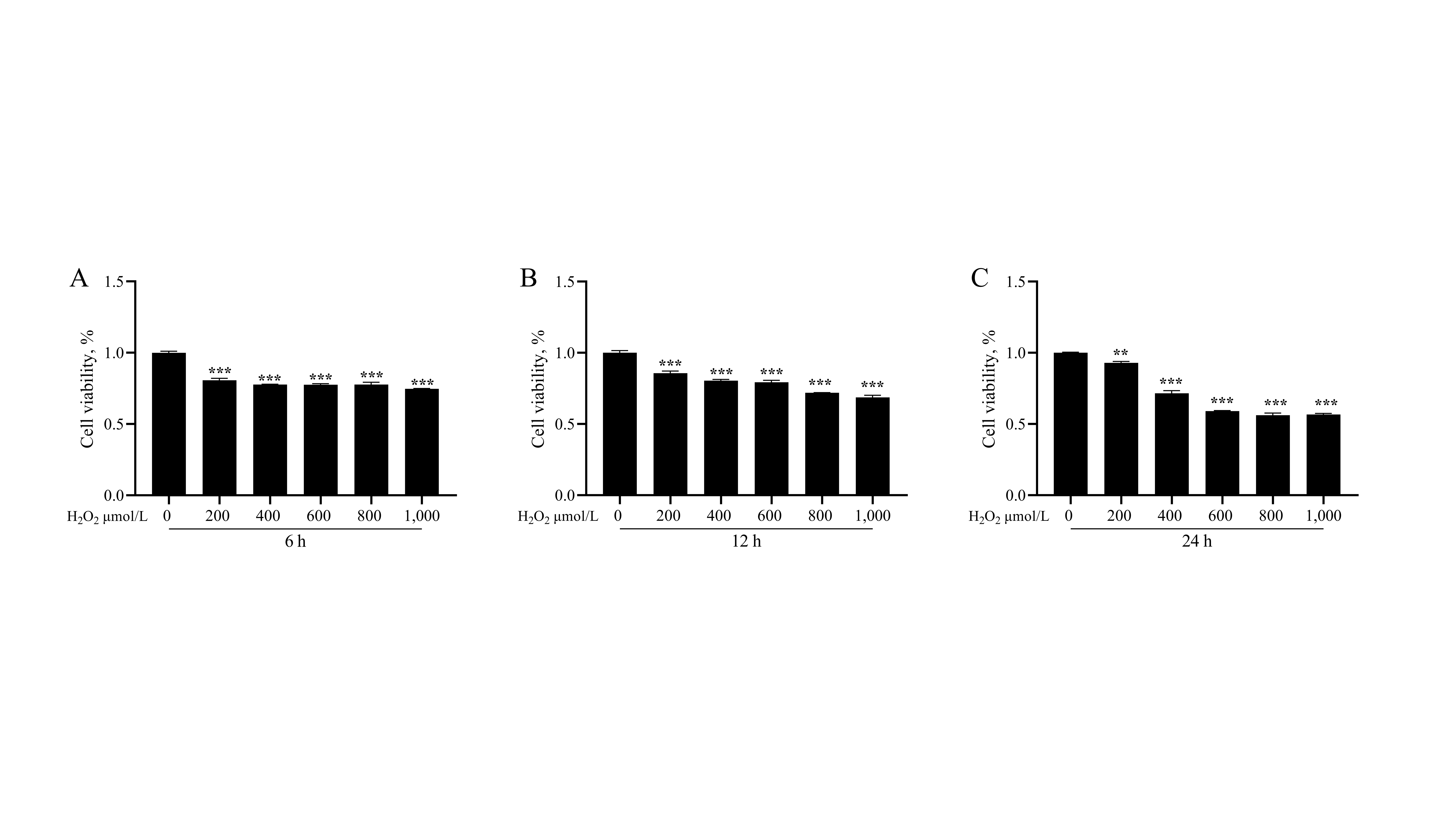

Supplement: Supplementary file 1 — Additional file 1: Fig. S1. Effects of H2O2 on the viability of bMECs. A bMECs were treated with H2O2 (0, 200, 400, 600, 800, 1,000 μmol/L) for 6 h. B bMECs were treated with H2O2 (0, 200, 400, 600, 800, 1,000 μmol/L) for 12 h. C bMECs were treated with H2O2 (0, 200, 400, 600, 800, 1,000 μmol/L) for 24 h. All data are presented as the mean ± SEM from three independent experiments. *P< 0.05, **P< 0.01, and ***P< 0.001. [file 40104_2024_1012_MOESM1_ESM.tif]

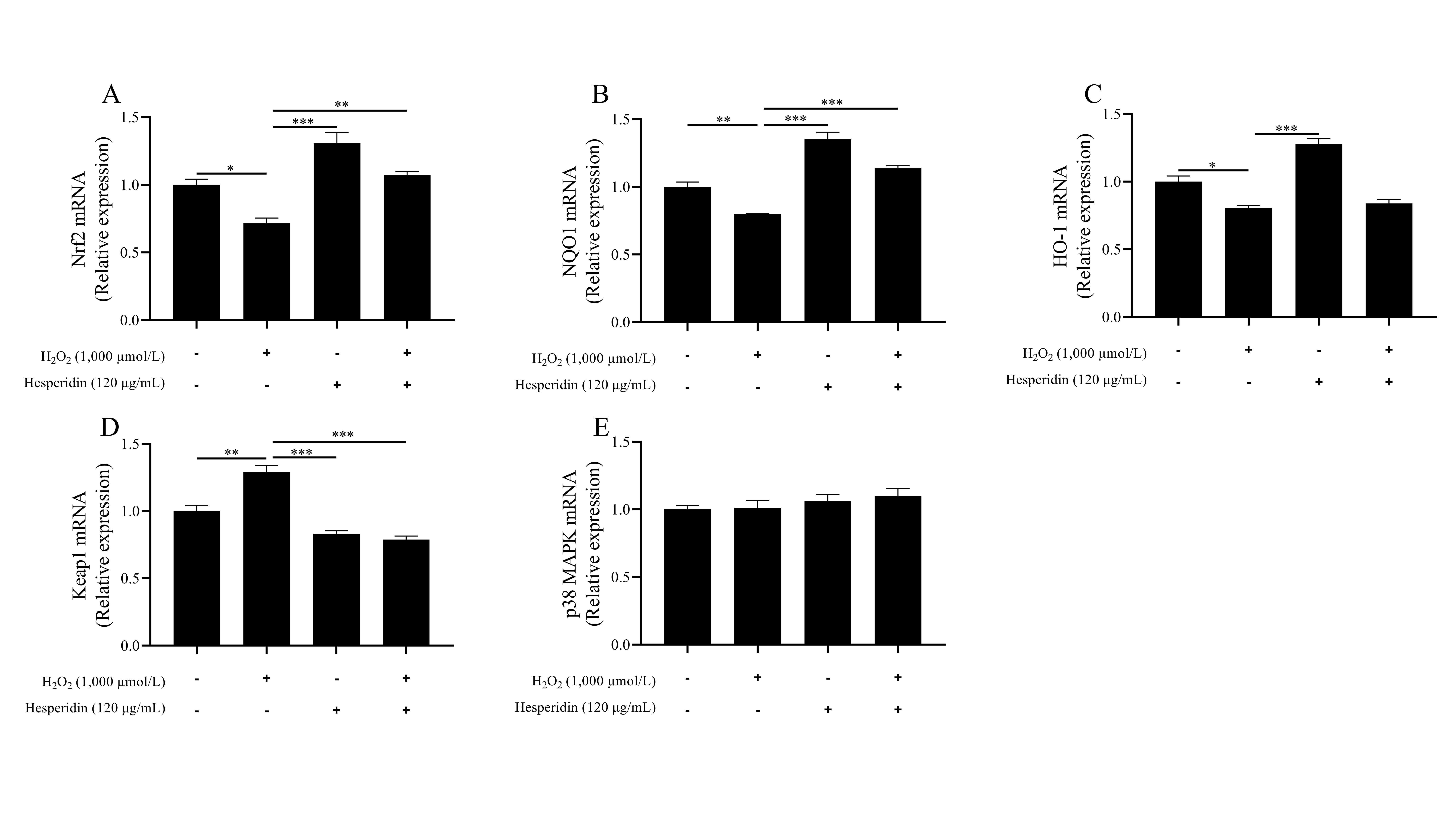

Supplement: Supplementary file 2 — Additional file 2: Fig. S2. Hesperidin induces the nuclear translocation of Nrf2 and activates the mRNA expression of antioxidant response genes in H2O2-treated bMECs. A Nrf2 mRNA expression. B NQO1 mRNA expression. C HO-1 mRNA expression. D Keap1 mRNA expression. E p38 MAPK mRNA expression. bMECs were treated with H2O2 (1,000 μmol/L) for 8 h and/or hesperidin (120 μg/mL) for 24 h. All data are presented as the mean ± SEM from three independent experiments. *P< 0.05, **P< 0.01, and ***P< 0.001. [file 40104_2024_1012_MOESM2_ESM.tif]
